# Crystal structure of glycidamide: the mutagenic and genotoxic metabolite of acryl­amide

**DOI:** 10.1107/S2056989016010859

**Published:** 2016-07-22

**Authors:** Melanie N. Hemgesberg, Thorsten Bonck, Karl-Heinz Merz, Yu Sun, Dieter Schrenk

**Affiliations:** a Food Chemistry and Toxicology, University of Kaiserslautern, 67663 Kaiserslautern, Germany; b Theoretical Chemistry, University of Kaiserslautern, 67663 Kaiserslautern, Germany; c Inorganic Chemistry, University of Kaiserslautern, 67663 Kaiserslautern, Germany

**Keywords:** crystal structure, glycidamide, acryl­amide, mutagenic, genotoxic metabolite, hydrogen bonding, β-sheet

## Abstract

The title compound, glycidamide (systematic name: oxirane-2-carboxamide), C_3_H_5_NO_2_, is the mutagenic and genotoxic metabolite of acryl­amide, a food contaminant and industrial chemical that has been classified as being probably carcinogenic to humans. Synthesized *via* the reaction of acrylo­nitrile and hydrogen peroxide, it crystallizes with both enanti­omers occurring as two crystallographically independent mol­ecules (*A* and *B*) in the asymmetric unit. They have similar conformations with an r.m.s. deviation of 0.0809 Å for mol­ecule *B* inverted on mol­ecule *A*. In the crystal, mol­ecules are linked by N—H⋯O hydrogen bonds, which lead to the formation of β-sheet structures enclosing *R*_2_^2^(8) and *R*_4_^2^(8) loops. The β-sheets are linked by weaker C—H⋯O hydrogen bonds, forming a supra­molecular three-dimensional structure.

## Abstract 2

The title compound, glycidamide, was synthesized *via* the reaction of acrylo­nitrile and hydrogen peroxide. Both enanti­omers occur as two crystallographically independent mol­ecules in the asymmetric unit. In the crystal, mol­ecules are linked by N—H⋯O hydrogen bonds forming β-sheet structures. The β-sheets are linked by weaker C—H⋯O hydrogen bonds, forming a supra­molecular three-dimensional structure.

## Chemical context   

The formation of glycidamide (GA) is considered to cause the carcinogenicity of acryl­amide (AA; Udovenko & Kolzunova, 2008 ▸), which is a widely used chemical in industry (EPA, 1994 ▸). Typical applications include the production of copolymers, flocculation agents and carrier material for gel electrophoresis. Moreover, it is formed if certain foods are heated to temperatures above 393 K at low moisture. AA was found at the highest levels in solid coffee substitutes, fried potato products and gingerbread, thus contributing to human exposure (EFSA, 2015 ▸). AA forms predominantly from asparagine in the presence of reducing sugars during the Maillard reaction *via* a Strecker-type degradation (Mottram *et al.*, 2002 ▸; Stadler *et al.*, 2002 ▸; Tareke *et al.*, 2002 ▸; Yaylayan *et al.*, 2003 ▸). Besides being a food contaminant, AA is also a component of tobacco smoke (Papoušek *et al.*, 2014 ▸). It has also been classified as ‘probably carcinogenic to humans (Group 2A)’ by the Inter­national Agency for Research on Cancer (IARC, 1994 ▸). It has not been found to be mutagenic or genotoxic without metabolic activation to GA at biologically relevant concentrations (Watzek *et al.*, 2012 ▸).
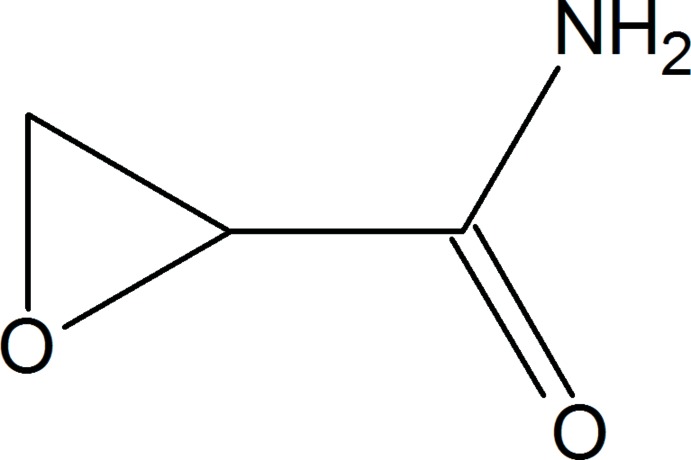


GA is a genotoxic and mutagenic compound formed *in vivo* metabolically from AA, mainly in the liver by cytochrome P450 2E1 (Baum *et al.*, 2005 ▸). As a reactive epoxide, GA is able to react with nucleophilic centers of proteins and DNA, thus forming DNA adducts and hemoglobin conjugates (Ghanayem *et al.*, 2005 ▸). As a consequence, mutations may occur, which represent stages of chemical mutagenesis and carcinogenesis (Gamboa da Costa *et al.*, 2003 ▸). We synthesized GA *via* the reaction of acrylo­nitrile and hydrogen peroxide.

## Structural commentary   

**Figure 1:**
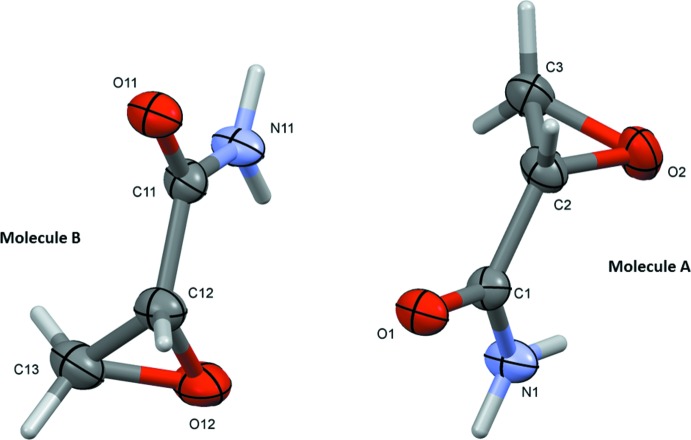
The mol­ecular structure of the two independent mol­ecules (*A* and *B*) of the title compound, glycidamide (GA), with atom labelling. Displacement ellipsoids are drawn at the 50% probability level.

Owing to its size, GA shows few structural features. Both enanti­omers occur as two crystallographically independent mol­ecules (*A* and *B*) in the asymmetric unit (Fig. 1 ▸). They have similar conformations with an r.m.s. deviation of 0.0809 Å for mol­ecule *B* inverted on mol­ecule *A*. The amide group is inclined to the epoxide plane by 77.9 (2)° in mol­ecule *A* (N1/C1/O1 *vs* O2/C2/C3), and by 72.6 (2)° in mol­ecule *B* (N11/C11/O11 *vs* O12/C12/C13).

Of inter­est are the C—C as well as the C—O bond lengths in the epoxide fragment (Table 1 ▸). The values of bond lengths in the epoxide fragments of both enanti­omers are compared to the mean values and their standard deviation of a selection of 149 similarly substituted compounds featuring an epoxide fragment (Table 1 ▸), which were reported to the Cambridge Structural Database (CSD, Version 5.37, Update 2 Feb 2016; Groom *et al.*, 2016 ▸). While the epoxide C—O bonds in GA match the mean values quite well, the C—C bond is more at the upper end for bond lengths. Nevertheless, the C—C bond length is still in the range of one standard deviation to the mean value of the database entries. Omitting oxirane itself, GA is the smallest example of an epoxide crystal structure reported in the CSD. Summing up, the epoxide fragment in GA seems to be representative for this class of epoxides.

## Supra­molecular features   

**Figure 2:**
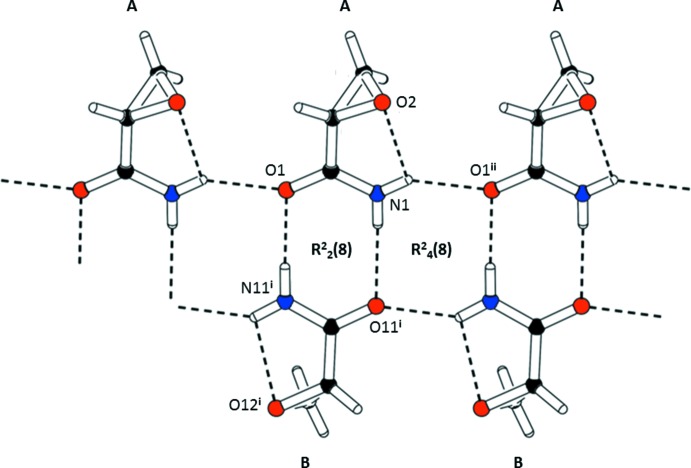
A partial view of the crystal packing of the title compound, showing the β-sheet arrangement, formed through strong N—H⋯O hydrogen bonds (dashed lines; see Table 2 ▸ for details), propagating along the *b-*axis direction.

**Figure 3:**
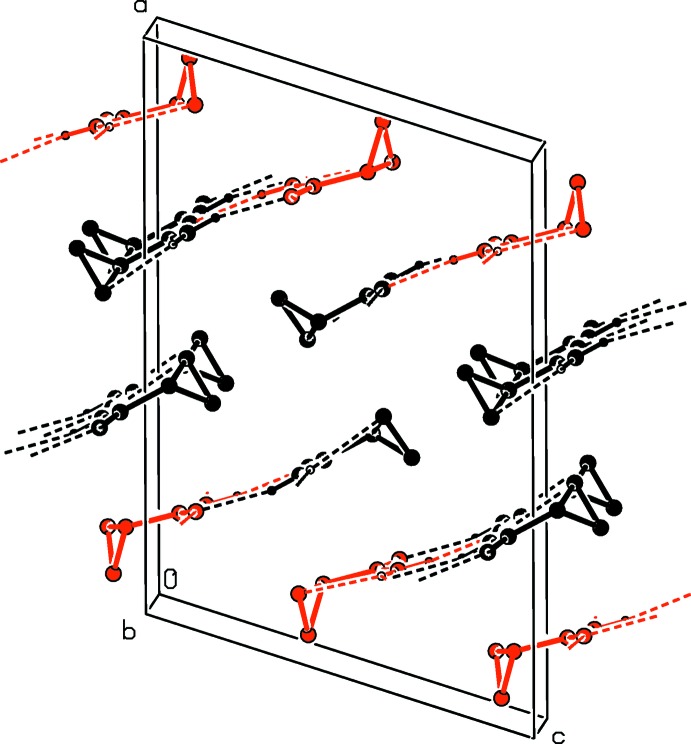
A view along the *b* axis of the crystal packing of the title compound, showing the β-sheet arrangement formed through strong N—H⋯O hydrogen bonds (dashed lines; see Table 2 ▸ for details). The C-bound H atoms have been omitted for clarity (*A* mol­ecules = black; *B* mol­ecules = red).

**Figure 4:**
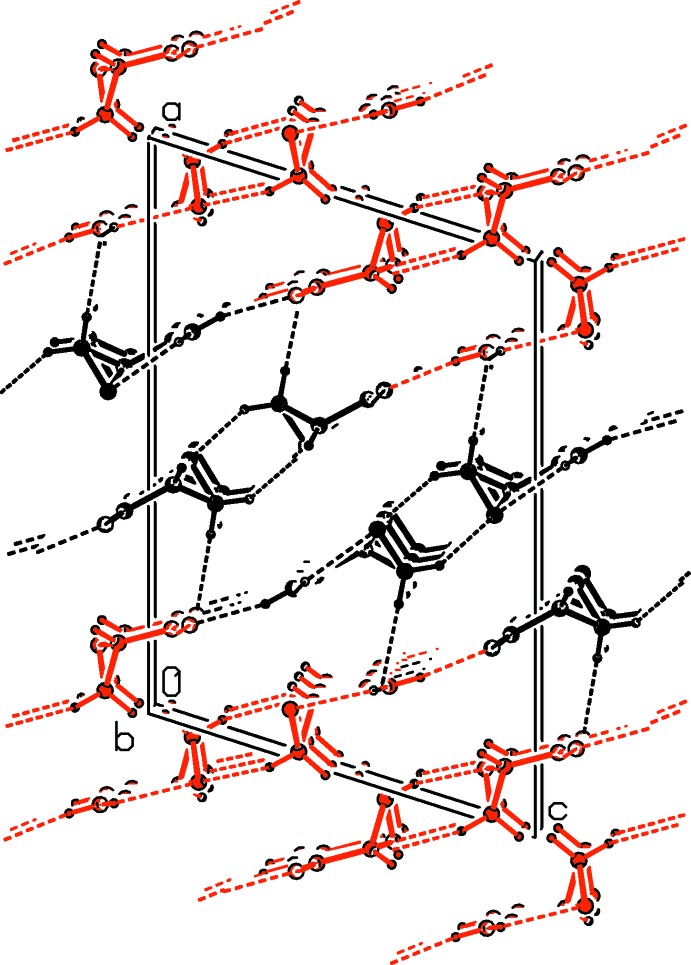
A view along the *b* axis of the crystal packing of the title compound. The N—H⋯O and C—H⋯O hydrogen bonds are shown as dashed lines (see Table 2 ▸ for details). H atoms not involved in these inter­actions have been omitted for clarity (*A* mol­ecules = black; *B* mol­ecules = red).

In the crystal, there are as expected, hydrogen bonds dominating the solid-state structure. The protons of the amino moiety undergo strong N—H⋯O hydrogen bonding to the carbonyl groups of adjacent GA mol­ecules (Table 2 ▸, Fig. 2 ▸). This results in the formation of a β-sheet structure, which is parallel to the crystallographic *b* axis and encloses 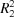
(8) and 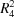
(8) loops. The β-sheets are also oriented parallel to each other (Fig. 3 ▸). They are further inter­linked by additional but weaker C—H⋯O hydrogen bonds (Table 2 ▸), between the protons ot the –CH_2_– units with the carbonyl group and the ep­oxy function from the neighbouring β-sheets, which leads to the formation of a supra­molecular three-dimensional structure (Fig. 4 ▸).

## Database survey   

As noted in Section 2, a search of the Cambridge Structural Database (Groom *et al.*, 2016 ▸) revealed the presence of 149 similarly substituted compounds featuring an epoxide fragment. However, up to now there has been no report of the structure of the title compound (GA).

## Synthesis and crystallization   

The synthesis of the title compound (GA) was performed according to a published method with modifications (Payne & Williams, 1961 ▸). The conventional literature procedure by controlled pH and temperature resulted in an unfavorable decomposition of hydrogen peroxide. GA was synthesized by dropwise addition of 1 *M* NaOH (60 ml) to acrylo­nitrile (80.1 g, 1.22 mol) in water (500 ml) and 30% H_2_O_2_ (102 ml, 1 mol). The pH was kept at 7.3–7.5 and the temperature was maintained at 308–310 K. After the reaction was completed (about 12 h), the mixture was treated with 5% palladium on charcoal, stored overnight in a refrigerator and then filtered. The solvent was evaporated and the crude product (yield: 55 g; 63%) was recrystallized from dry acetone at low temperature. Colourless crystals formed after 3–5 days at 243 K. GA is very hygroscopic, so purification of the raw product was carried out in an inert atmosphere. The compound was stored in dry argon at 243 K. Identity and purity were checked by NMR spectroscopic methods and elemental analysis. ^1^H-NMR (600.13 MHz, 295.15 K, p.p.m., D_2_O): δ 3,49 (*dd*, ^2^ *J*_HH_ = 4.08 Hz, ^3^ *J*_HH_ = 2.58 Hz,1H); 3,02 (*t*, 5.16 Hz, 1H); 2,87 (*dd*, ^2^ *J*_HH_ = 5.52 Hz, ^3^ *J*_HH_ = 2.58 Hz, 1H). ^13^C-{^1^H}-NMR (100.66 MHz, 294.05 K, p.p.m., DMSO-*d*_6_): δ 170.1 (C1), 48.5 (C2), 45.6 (C3). Elemental analysis for C_3_H_5_NO_2_. Required: C 41.36%; H 5.79%; N 16.09%; found: C 41.41%; H 5.47%; N 16.27%.

## Refinement   

Crystal data, data collection and structure refinement details are summarized in Table 3 ▸. The H atoms bound to the nitro­gen atoms, N1 and N11, were located in a difference Fourier map, and refined with a distance restraint: N—H = 0.86 (2) Å with *U*_iso_(H) = 1.2*U*_eq_(N). The C-bound H were placed in calculated positions and refined using a riding model: C—H = 0.99–1.00 Å with *U*_iso_(H) = 1.2*U*_eq_(C).

## Supplementary Material

Crystal structure: contains datablock(s) I, Global. DOI: 10.1107/S2056989016010859/su5309sup1.cif

Structure factors: contains datablock(s) I. DOI: 10.1107/S2056989016010859/su5309Isup2.hkl

Supporting information file. DOI: 10.1107/S2056989016010859/su5309Isup3.cml

CCDC reference: 1490160

Additional supporting information: crystallographic information; 3D view; checkCIF report

## Figures and Tables

**Table 1:** Experimental bond lengths (Å) compared to a database survey of 149 compounds featuring epoxide fragments

	Bond	Mol­ecule *A*	Bond	Mol­ecule *B*	Database survey
C—C	C2—C3	1.463 (2)	C12—C13	1.458 (2)	1.442±0.028
CH_2_—O	C3—O2	1.436 (2)	C13—O12	1.433 (2)	1.431±0.026
*X*CH—O	C2—O2	1.429 (2)	C12—O12	1.424 (2)	1.432±0.026

**Table 2:** Hydrogen-bond geometry (Å, °)

*D*—H⋯*A*	*D*—H	H⋯*A*	*D*⋯*A*	*D*—H⋯*A*
N1—H1*B*⋯O2	0.86 (2)	2.45 (2)	2.7770 (17)	103 (1)
N11—H11*B*⋯O12	0.87 (2)	2.39 (2)	2.7408 (17)	105 (1)
N1—H1*A*⋯O11^i^	0.86 (2)	2.12 (2)	2.9651 (16)	167 (2)
N1—H1*B*⋯O1^ii^	0.86 (2)	2.12 (2)	2.8482 (14)	142 (2)
N11—H11*A*⋯O1^iii^	0.87 (2)	2.08 (2)	2.9447 (16)	173 (2)
N11—H11*B*⋯O11^ii^	0.87 (2)	2.11 (2)	2.8495 (14)	144 (2)
C3—H3*A*⋯O11^ii^	0.99	2.59	3.5839 (19)	179
C3—H3*B*⋯O2^iv^	0.99	2.59	3.4470 (18)	144
C13—H13*A*⋯O12^v^	0.99	2.44	3.3991 (19)	163

Symmetry codes: (i) 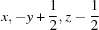; (ii) 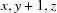; (iii) 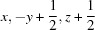; (iv) 
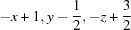
; (v) 
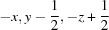
.

**Table 3:** Experimental details

Crystal data	
Chemical formula	C_3_H_5_NO_2_
*M*_r_	87.08
Crystal system, space group	Monoclinic, *P*2_1_/*c*
Temperature (K)	150
*a*, *b*, *c* (Å)	15.5186 (7), 5.1007 (2), 10.9250 (5)
β (°)	107.651 (5)
*V* (Å^3^)	824.06 (7)
*Z*	8
Radiation type	Cu *K*α
μ (mm^−1^)	1.02
Crystal size (mm)	0.22 × 0.16 × 0.16

Data collection	
Diffractometer	Rigaku Xcalibur (Sapphire3, Gemini ultra)
Absorption correction	Multi-scan (*CrysAlis PRO*; Rigaku Oxford Diffraction, 2015 ▸)
*T*_min_, *T*_max_	0.837, 1.000
No. of measured, independent and observed [*I* > 2σ(*I*)] reflections	4485, 1310, 1207
*R*_int_	0.022
(sin θ/λ)_max_ (Å^−1^)	0.577

Refinement	
*R*[*F* ^2^ > 2σ(*F* ^2^)], *wR*(*F* ^2^), *S*	0.035, 0.093, 1.10
No. of reflections	1310
No. of parameters	121
No. of restraints	4
H-atom treatment	H atoms treated by a mixture of independent and constrained refinement
Δρ_max_, Δρ_min_ (e Å^−3^)	0.34, −0.17

Computer programs: *CrysAlis PRO* (Rigaku Oxford Diffraction, 2015 ▸), *SIR2014* (Burla *et al.*, 2015 ▸), *SHELXL2014* (Sheldrick, 2015 ▸), *Mercury* (Macrae *et al.*, 2008 ▸) and *PLATON* (Spek, 2009 ▸).

## supplementary crystallographic information

### Crystal data

**Table:**

C_3_H_5_NO_2_	*F*(000) = 368
*M**_r_* = 87.08	*D*_x_ = 1.404 Mg m^−^^3^
Monoclinic, *P*2_1_/*c*	Cu *K*α radiation, λ = 1.54184 Å
*a* = 15.5186 (7) Å	Cell parameters from 2255 reflections
*b* = 5.1007 (2) Å	θ = 6.0–62.6°
*c* = 10.9250 (5) Å	µ = 1.02 mm^−^^1^
β = 107.651 (5)°	*T* = 150 K
*V* = 824.06 (7) Å^3^	Transparent prism, colorless
*Z* = 8	0.22 × 0.16 × 0.16 mm

### Data collection

**Table:**

Rigaku Xcalibur (Sapphire3, Gemini ultra) diffractometer	1310 independent reflections
Radiation source: fine-focus sealed X-ray tube	1207 reflections with *I* > 2σ(*I*)
Detector resolution: 16.1399 pixels mm^-1^	*R*_int_ = 0.022
ω scans	θ_max_ = 62.8°, θ_min_ = 6.0°
Absorption correction: multi-scan (CrysAlis PRO; Rigaku Oxford Diffraction, 2015)	*h* = −17→14
*T*_min_ = 0.837, *T*_max_ = 1.000	*k* = −5→5
4485 measured reflections	*l* = −8→12

### Refinement

**Table:**

Refinement on *F*^2^	Primary atom site location: structure-invariant direct methods
Least-squares matrix: full	Secondary atom site location: difference Fourier map
*R*[*F*^2^ > 2σ(*F*^2^)] = 0.035	Hydrogen site location: difference Fourier map
*wR*(*F*^2^) = 0.093	H atoms treated by a mixture of independent and constrained refinement
*S* = 1.10	*w* = 1/[σ^2^(*F*_o_^2^) + (0.0458*P*)^2^ + 0.238*P*] where *P* = (*F*_o_^2^ + 2*F*_c_^2^)/3
1310 reflections	(Δ/σ)_max_ < 0.001
121 parameters	Δρ_max_ = 0.34 e Å^−^^3^
4 restraints	Δρ_min_ = −0.17 e Å^−^^3^

### Special details

**Table:**

Geometry. All esds (except the esd in the dihedral angle between two l.s. planes) are estimated using the full covariance matrix. The cell esds are taken into account individually in the estimation of esds in distances, angles and torsion angles; correlations between esds in cell parameters are only used when they are defined by crystal symmetry. An approximate (isotropic) treatment of cell esds is used for estimating esds involving l.s. planes.

### Fractional atomic coordinates and isotropic or equivalent isotropic displacement parameters (Å^2^)

**Table:**

	*x*	*y*	*z*	*U*_iso_*/*U*_eq_	
C1	0.34078 (9)	0.3918 (3)	0.43936 (13)	0.0242 (3)	
C2	0.41336 (9)	0.3997 (3)	0.56563 (14)	0.0292 (4)	
H2	0.4507	0.2371	0.5900	0.035*	
C3	0.40088 (10)	0.5531 (3)	0.67215 (13)	0.0342 (4)	
H3A	0.3427	0.6464	0.6580	0.041*	
H3B	0.4285	0.4861	0.7604	0.041*	
N1	0.31052 (9)	0.6180 (2)	0.38512 (12)	0.0303 (3)	
H1A	0.2683 (11)	0.620 (4)	0.3127 (15)	0.036*	
H1B	0.3322 (11)	0.766 (3)	0.4175 (16)	0.036*	
O1	0.31121 (7)	0.17614 (19)	0.39333 (9)	0.0300 (3)	
O2	0.46035 (7)	0.6420 (2)	0.60247 (10)	0.0361 (3)	
C11	0.16824 (9)	0.1061 (3)	0.56672 (13)	0.0262 (3)	
C12	0.11401 (10)	0.1195 (3)	0.42784 (14)	0.0320 (4)	
H12	0.1266	−0.0195	0.3709	0.038*	
C13	0.02176 (11)	0.2199 (3)	0.39198 (15)	0.0392 (4)	
H13A	−0.0228	0.1437	0.3153	0.047*	
H13B	−0.0030	0.2724	0.4618	0.047*	
N11	0.18834 (8)	0.3303 (2)	0.62832 (12)	0.0294 (3)	
H11A	0.2208 (11)	0.332 (4)	0.7085 (14)	0.035*	
H11B	0.1752 (11)	0.480 (3)	0.5899 (16)	0.035*	
O11	0.18951 (7)	−0.11167 (18)	0.61662 (9)	0.0323 (3)	
O12	0.09383 (8)	0.3738 (2)	0.37277 (11)	0.0441 (3)	

### Atomic displacement parameters (Å^2^)

**Table:**

	*U*^11^	*U*^22^	*U*^33^	*U*^12^	*U*^13^	*U*^23^
C1	0.0286 (7)	0.0184 (8)	0.0250 (7)	−0.0003 (5)	0.0071 (5)	−0.0010 (5)
C2	0.0280 (7)	0.0245 (8)	0.0313 (7)	−0.0010 (6)	0.0032 (6)	−0.0015 (6)
C3	0.0337 (7)	0.0403 (9)	0.0263 (7)	−0.0070 (7)	0.0060 (6)	−0.0045 (6)
N1	0.0408 (7)	0.0163 (7)	0.0266 (6)	−0.0025 (5)	−0.0004 (5)	−0.0008 (5)
O1	0.0388 (6)	0.0157 (5)	0.0298 (5)	−0.0012 (4)	0.0017 (4)	−0.0015 (4)
O2	0.0330 (6)	0.0383 (6)	0.0358 (6)	−0.0118 (4)	0.0084 (4)	−0.0103 (5)
C11	0.0281 (7)	0.0191 (8)	0.0286 (7)	−0.0011 (5)	0.0044 (6)	0.0009 (5)
C12	0.0384 (8)	0.0253 (8)	0.0279 (7)	−0.0024 (6)	0.0035 (6)	−0.0002 (5)
C13	0.0364 (8)	0.0361 (9)	0.0363 (8)	−0.0010 (7)	−0.0022 (6)	0.0007 (7)
N11	0.0377 (7)	0.0162 (6)	0.0265 (6)	−0.0004 (5)	−0.0022 (5)	0.0021 (5)
O11	0.0411 (6)	0.0171 (6)	0.0317 (5)	0.0005 (4)	0.0008 (4)	0.0006 (4)
O12	0.0484 (7)	0.0370 (7)	0.0364 (6)	−0.0047 (5)	−0.0027 (5)	0.0135 (5)

### Geometric parameters (Å, º)

**Table:**

C1—O1	1.2383 (17)	C11—O11	1.2373 (17)
C1—N1	1.3169 (18)	C11—N11	1.3154 (19)
C1—C2	1.494 (2)	C11—C12	1.497 (2)
C2—O2	1.4294 (17)	C12—O12	1.4242 (19)
C2—C3	1.463 (2)	C12—C13	1.458 (2)
C2—H2	1.0000	C12—H12	1.0000
C3—O2	1.4362 (19)	C13—O12	1.433 (2)
C3—H3A	0.9900	C13—H13A	0.9900
C3—H3B	0.9900	C13—H13B	0.9900
N1—H1A	0.860 (15)	N11—H11A	0.868 (15)
N1—H1B	0.856 (15)	N11—H11B	0.864 (15)
			
O1—C1—N1	123.91 (12)	O11—C11—N11	124.39 (12)
O1—C1—C2	118.80 (12)	O11—C11—C12	118.71 (12)
N1—C1—C2	117.26 (12)	N11—C11—C12	116.89 (12)
O2—C2—C3	59.53 (9)	O12—C12—C13	59.62 (10)
O2—C2—C1	117.44 (12)	O12—C12—C11	116.98 (12)
C3—C2—C1	120.29 (12)	C13—C12—C11	119.53 (14)
O2—C2—H2	115.9	O12—C12—H12	116.2
C3—C2—H2	115.9	C13—C12—H12	116.2
C1—C2—H2	115.9	C11—C12—H12	116.2
O2—C3—C2	59.07 (9)	O12—C13—C12	59.02 (10)
O2—C3—H3A	117.9	O12—C13—H13A	117.9
C2—C3—H3A	117.9	C12—C13—H13A	117.9
O2—C3—H3B	117.9	O12—C13—H13B	117.9
C2—C3—H3B	117.9	C12—C13—H13B	117.9
H3A—C3—H3B	115.0	H13A—C13—H13B	115.0
C1—N1—H1A	119.5 (12)	C11—N11—H11A	119.9 (12)
C1—N1—H1B	122.9 (12)	C11—N11—H11B	122.2 (12)
H1A—N1—H1B	117.6 (17)	H11A—N11—H11B	117.5 (17)
C2—O2—C3	61.40 (10)	C12—O12—C13	61.35 (10)

### Hydrogen-bond geometry (Å, º)

**Table:**

*D*—H···*A*	*D*—H	H···*A*	*D*···*A*	*D*—H···*A*
N1—H1*B*···O2	0.86 (2)	2.45 (2)	2.7770 (17)	103 (1)
N11—H11*B*···O12	0.87 (2)	2.39 (2)	2.7408 (17)	105 (1)
N1—H1*A*···O11^i^	0.86 (2)	2.12 (2)	2.9651 (16)	167 (2)
N1—H1*B*···O1^ii^	0.86 (2)	2.12 (2)	2.8482 (14)	142 (2)
N11—H11*A*···O1^iii^	0.87 (2)	2.08 (2)	2.9447 (16)	173 (2)
N11—H11*B*···O11^ii^	0.87 (2)	2.11 (2)	2.8495 (14)	144 (2)
C3—H3*A*···O11^ii^	0.99	2.59	3.5839 (19)	179
C3—H3*B*···O2^iv^	0.99	2.59	3.4470 (18)	144
C13—H13*A*···O12^v^	0.99	2.44	3.3991 (19)	163

Symmetry codes: (i) *x*, −*y*+1/2, *z*−1/2; (ii) *x*, *y*+1, *z*; (iii) *x*, −*y*+1/2, *z*+1/2; (iv) −*x*+1, *y*−1/2, −*z*+3/2; (v) −*x*, *y*−1/2, −*z*+1/2.
